# Supplementary material for: A Review of the Current Status of G6PD Deficiency Testing to Guide Radical Cure Treatment for Vivax Malaria
Source: Pathogens. 2023 Apr 27;12(5):650. doi: 10.3390/pathogens12050650 (PMC10220632; doi:10.3390/pathogens12050650)
Supplement: Supplementary file 1 [file pathogens-12-00650-s001.zip › pathogens-2342386-supplementary/G6PD Dx Rev_suppl_Table S1_Ref updated.docx]

Supplementary Information to A review of the current status of G6PD deficiency testing to guide radical cure treatment for vivax malaria

**Table S1.** Radical cure and G6PD testing policy in countries in the Asia-Pacific.

| **Country** | **Last Policy Update** | **Policy on G6PD testing** | **Definition of 100% Activity** | **Categorization of G6PD Status Used** | **Policy on Primaquine Treatment** | **Additional Recommendation(s) to Ensure Safety** | **Implementation of G6PD Testing** |
| --- | --- | --- | --- | --- | --- | --- | --- |
| Afghanistan [80,81] | 2017 | Recommended,  quantitative for  females | Not specified | Normal:  > 80%  enzyme  activity | PQ 0.25 mg/kg over 14 days | No  information | Testing not  implemented  (Personal communication - Dr. Naeem, Malaria program) |
|  |  |  |  | Intermediate:  30–80%  enzyme  activity | PQ 0.75 mg/kg weekly for 8 weeks | Close  medical  supervision |  |
|  |  |  |  | Deficient:  < 30%  enzyme  activity | No PQ |  |  |
|  |  |  |  | If no testing  available | PQ 0.75 mg/kg weekly for 8 weeks | Close  medical  supervision |  |
| Bangladesh [82,83] | 2017 | Not explicitly  mentioned. | Not specified | Not  specified | PQ 0.25 mg/kg over 14 days | Close  medical  supervision and  referral for  management of  severe conditions  Counselling for adverse events | Quantitative testing using STANDARD G6PD rolled out in a few high-risk areas in 2022. GF supported. Policy to be updated 2023 to add quantitative G6PD testing as recommendation before vivax treatment. (Personal  communication - Ekramul Haque, Malaria Program) |
| Bhutan [84,85] | 2019 | Recommended but test type not specified | Not specified | All  uncomplicated vivax cases | PQ 0.25 mg/kg over 14 days | Close  medical  supervision and  patient  counselling | Rollout of STANDARD G6PD planned for 2023 (date not specified). HW already trained in 13 risk areas for STANDARD G6PD (Personal  communication - Dr. Tobgyel, Ministry of Health, Bhutan) |
|  |  |  |  | Deficient | Not explicitly mentioned |  |  |
|  |  |  |  | If no testing  available | Based on risk-benefit  assessment |  |  |
| Cambodia [86] | 2014 | Not explicitly  mentioned | Not specified | Normal  (no definition) | PQ 0.25 mg/kg over 14 days or PQ 0.75 mg/kg weekly for 8 weeks | Counselling for adverse events | Testing implemented in all malaria-endemic districts using STANDARD G6PD. GF funded. (Dr. Dysoley Lek, Malaria program) |
|  |  |  |  | Deficient  (no definition) | No PQ | NA |  |
|  |  |  |  | If no testing  available | Not specified |  |  |
| India [87–89] | 2016 | Recommended but test type not specified | Not specified | Normal  (no definition) | PQ 0.25 mg/kg over 14 days | Under  medical supervision; counselling for adverse events | Testing not  implemented in the public health facilities  (Personal communication - Dr. Neena- former WHO SEARO, Abhijit Sharma, PATH) |
|  |  |  |  | Deficient  (no definition) | No PQ | NA |  |
|  |  |  |  | If no testing  available | Not specified |  |  |
| Indonesia [90] | 2020 | Not explicitly  mentioned  (required for relapsed cases) | Not specified | Normal  (no definition) | PQ 0.25 mg/kg over 14 days  PQ 0.5 mg/kg over 14 days (relapse) | No information | STANDARD G6PD rollout in context of PAVE  studies (Personal  communication –  Dr Ayodhia Pitaloka Pasaribu,  University  Sumatera Utara) |
|  |  |  |  | Deficient  (no definition) | No PQ | NA |  |
|  |  |  |  | If no testing  available | Not specified |  |  |
| Lao PDR [91–93] | 2022 | Recommended,  quantitative | Not specified | Normal  Males:  > 4 IU/g Hb  Females:  > 6 IU/g Hb | PQ 0.5 mg/kg  over 7 days | Counselling for adverse events | Testing implemented using  STANDARD G6PD country-wide.  GF funded (Personal communication –  Dr. Boualam Khamlome, Malaria Program) |
|  |  |  |  | Intermediate  females:  4–6 IU/g Hb | PQ 0.75 mg/kg weekly for 8 weeks | Counselling for adverse events |  |
|  |  |  |  | Deficient:  < 3.9 IU/g Hb | PQ 0.75 mg/kg weekly for 8 weeks | Counselling for adverse events |  |
|  |  |  |  | If no testing  available | PQ 0.75 mg/kg weekly for 8 weeks | Counselling for adverse events |  |
| Myanmar [94,95] | 2015 | Recommended but test type not specified | Not specified | Normal  (no definition) | PQ 0.25 mg/kg over 14 days | Counselling for adverse events | Testing using STANDARD G6PD is only done in areas where SMRU works. GF funded (Personal communication with Dr. L Zau Ring/NMCP and Dr. Aung Myint Thu/SMRU - |
|  |  |  |  | Mild to moderate (no definition) | PQ 0.75 mg/kg weekly for 8 weeks | Counselling for adverse events |  |
|  |  |  |  | Severe  Deficient  (no definition) | No PQ | NA |  |
|  |  |  |  | If no testing  available | Risk-benefit assessment | Not specified |  |
| Nepal [96,97] | 2019 | Recommended but test type not specified | Not specified | Normal  (no definition) | PQ 0.25 mg/kg over 14 days | Under medical supervision  Counselling for adverse events | Testing not implemented (Personal communication with Ram Kumar Mahato, National Malaria Program) |
|  |  |  |  | Deficient  (no definition) | Not specified |  |  |
|  |  |  |  | If no testing  available | PQ 0.25 mg/kg over 14 days |  |  |
| Pakistan [98,99] | 2018 | Recommended,  quantitative | Not specified | Normal  (no definition) | PQ 0.25 mg/kg over 14 days | Counselling for adverse events | Testing not implemented.  Policy revision underway to endorse quantitative testing  (Personal communication –  Dr. Zheeshan – Independent consultant) |
|  |  |  |  | Mild to moderate (no definition) | PQ 0.75 mg/kg  weekly for 8 weeks |  |  |
|  |  |  |  | Severe deficient | No PQ | NA |  |
|  |  |  |  | If no testing  available | PQ 0.25mg/kg over 14 days (maximum 15 mg) | Close medical supervision  Counselling for adverse events |  |
| Papua New Guinea [100,101] | 2009 | Not explicitly  mentioned | Not specified | Normal  (no definition) | PQ 0.25mg/kg over 14 days | Counselling for adverse events | STANDARD G6PD rollout in context of PAVE studies (Personal communication - Prof Leanne Robinson, Burnett Institute) |
|  |  |  |  | Severe deficiency (no definition) | No PQ |  |  |
|  |  |  |  | If no testing  available | Not specified |  |  |
| Philippines [102] | 2018 | Not explicitly  mentioned | Not specified | Normal  (no definition) | PQ 0.25mg/kg over 14 days | Close monitoring | Routinely done newborn screening for G6PD spectrophometry and/or FST.  PoC testing not implemented.  (Personal communication -Dr. Sheen Angelou) |
|  |  |  |  | Deficient  (no definition) | Based on risk-benefit assessment  PQ 0.75 mg/kg weekly for 8 weeks | Close medical supervision |  |
|  |  |  |  | If no testing  available | Not specified | Not specified |  |
| Solomon Islands [103,104] | 2018 | Not explicitly  mentioned | Not specified | Normal  (no definition) | PQ 0.25 mg/kg over 14 days | Counselling for adverse events | Testing implemented using STANDARD G6PD in provincial hospitals.  GF funded (Personal communication Dr. Lyndes, Malaria program) |
|  |  |  |  | Deficient  (no definition) | No PQ | NA |  |
|  |  |  |  | If no testing  available | No PQ |  |  |
| Republic of Korea (ROK) [105] | 2019 | Recommended but test type not specified | Not specified | Normal  (no definition) | Adults: PQ 0.25 mg/kg over 14 days  Pediatrics: 0.3 mg/kg over 14 days | Counselling for  relapse  if irregular  administration  or discontinued | Testing not implemented in public facilities except for very few vivax cases in referred hospitals (Personal communication - Dr. Derek Lee, Malaria Program) |
|  |  |  |  | Deficient  (no definition) | PQ 0.75 mg/kg weekly for 8 weeks |  |  |
|  |  |  |  | If no testing  available | Not specified | NA |  |
| Thailand [106] | 2019 | Required,  quantitative | Not specified | Normal:  > 6 U/gHb enzyme  activity  (> 70%) | TQ 300 mg | Counselling for adverse events | Testing implemented using STANDARD G6PD in health facilities.  GF funded  (Personal communication -Dr. Prayuth, Malaria program) |
|  |  |  |  | Intermediate:  4–6 U/g Hb  enzyme  activity (30–70%) | PQ 0.25 mg/kg over 14 days | With  precaution |  |
|  |  |  |  | Deficient:  < 4 U/g Hb  enzyme activity  (< 30%) | PQ 0.75 mg/kg weekly for 8 weeks | With periodic follow up |  |
|  |  |  |  | If no testing  available | Not specified | Counselling for adverse events |  |
| Vanuatu [107,108] | 2021 | Recommended,  qualitative | Not specified | Male  Normal  (no definition) | PQ 0.5 mg/kg over 14 days | Under medical supervision | Unable to ascertain information on implementation. |
|  |  |  |  | Female normal  (no definition) | PQ 0.25 mg/kg over 14 days |  |  |
|  |  |  |  | Deficient  (no definition) | No PQ | NA |  |
|  |  |  |  |  | PQ 0.75 mg/kg weekly for 8 weeks | Close medical supervision with access to blood transfusion |  |
|  |  |  |  | If no testing  available | PQ 0.25 mg/kg over 14 days | Close  medical  supervision |  |
| Vietnam [109,110] | 2020 | Recommended but test type not specified | Not specified | Normal:  > 70%  enzyme  activity | Normal: PQ 0.25 mg/kg over 14 days | Not specified | G6PD testing using STANDARD G6PD is rolled out in health facilities of three high-risk provinces.  GF funded  (Personal communication -Dr. Thang, Malaria Program) |
|  |  |  |  | Intermediate:  females 30–70% enzyme activity | PQ 0.25 mg/kg over 14 days | Close  monitoring, Proper counselling on AE and report to health facility |  |
|  |  |  |  | Deficient:  < 30%  enzyme  activity | PQ 0.75 mg/kg weekly for 8 weeks | Treat only in facilities capable of close monitoring, blood transfusion, and after patient counselling |  |
|  |  |  |  | If no testing  available | PQ (dosage not specified) | Close monitoring |  |

Treatment guidelines could not be found for Democratic People's Republic of Korea (DPRK). NA = not applicable. GF = Global Fund. PAVE = Partnership for Vivax Elimination.
