# Supplementary material for: A Review of the Current Status of G6PD Deficiency Testing to Guide Radical Cure Treatment for Vivax Malaria
Source: Pathogens. 2023 Apr 27;12(5):650. doi: 10.3390/pathogens12050650 (PMC10220632; doi:10.3390/pathogens12050650)
Supplement: Supplementary file 1 [file pathogens-12-00650-s001.zip › pathogens-2342386-supplementary/G6PD Dx Rev_suppl_Table S2_Ref updated.docx]

Supplementary Information to A review of the current status of G6PD deficiency testing to guide radical cure treatment for vivax malaria

**Table S2.** Radical cure and G6PD testing policy in selected vivax endemic countries Africa and the Middle East.

| **Country** | **Last Policy Update** | **Policy on G6PD Testing** | **Definition of 100% Activity** | **Categorization of G6PD Status** | **Policy on Primaquine Treatment** | **Additional recommendation(s) to Ensure Safety** | **Implementation of G6PD Testing** |
| --- | --- | --- | --- | --- | --- | --- | --- |
| Ethiopia [111] | 2022 | Not required  (not explicitly  recommended) | NA | NA | PQ 0.25 mg/kg over 14 days | Close  monitoring,  counselling on AE and report to health facility in case of AE | No testing  implemented  (Personal  communication –  Dr Tamiru Degaga, Arba Minch University) |
| Somalia [112] | 2016 | Recommended  but testing type not specified | Not specified | Normal  (no definition) | PQ 0.25 mg/kg over 14 days | Under medical supervision | Unable to ascertain information on implementation |
|  |  |  |  | Deficient  (no definition) | No PQ | NA |  |
|  |  |  |  | If no testing  available | Not specified | NA |  |
| Sudan [113] | 2017 | Not explicitly  mentioned | NA | Normal  (no definition) | Adults: PQ  15 mg/day for 14 days  Children:  0.25 mg/kg over 14 days | Not specified | Unable to ascertain information on implementation |
|  |  |  |  | Deficient  (no definition) | No PQ |  |  |
|  |  |  |  | If no testing  available | Not specified |  |  |
| Madagascar [114] | 2015 | Recommended  but testing type not specified | Not  specified | Normal  (no definition) | PQ 0.25 mg/kg over 14 days | Not specified | No testing implemented  (Personal communication - PvSTATEM consortium) |
|  |  |  |  | Moderate  deficiency  (no definition) | PQ 0.75 mg/kg weekly for 8 weeks |  |  |
|  |  |  |  | Severe deficiency (no definition) | No PQ |  |  |
|  |  |  |  | If no testing  available | Not specified |  |  |

Treatment guidelines could not be found for Eritrea, Djibouti, South Sudan and Yemen. NA = Not applicable. AE = Adverse event.
