# Supplementary material for: A Review of the Current Status of G6PD Deficiency Testing to Guide Radical Cure Treatment for Vivax Malaria
Source: Pathogens. 2023 Apr 27;12(5):650. doi: 10.3390/pathogens12050650 (PMC10220632; doi:10.3390/pathogens12050650)
Supplement: Supplementary file 1 [file pathogens-12-00650-s001.zip › pathogens-2342386-supplementary/G6PD Dx Rev_suppl_Table S3_Ref updated.docx]

Supplementary Information to A review of the current status of G6PD deficiency testing to guide radical cure treatment for vivax malaria

**Table S3.** Radical cure and G6PD testing policy in countries in Central and South America.

| **Country** | **Last  Policy Update** | **Policy on G6PD Testing** | **Definition of 100% Activity** | **Categorization of G6PD  Status** | **Policy on Primaquine Treatment** | **Additional Recommendation(S) to Ensure Safety** | **Implementation of G6PD Testing** |
| --- | --- | --- | --- | --- | --- | --- | --- |
| Bolivia [115] | 2013 | Not explicitly  mentioned | Not  specified | Not  specified | PQ 0.5 mg/kg over 7 days | Supervised treatment | Unable to ascertain information on implementation. |
| Brazil [116] | 2020 | PQ:  Recommended,  qualitative or  quantitative  TQ: Required,  quantitative | Not  specified | Normal  (no definition) | PQ 0.5 mg/kg/day over 7 days |  | STANDARD G6PD rollout  in context of feasibility studies  and implementation  in two municipalities with temporary  approval (review by CONITEC ongoing)  (Personal communication,  Jose Diego De Brito Sousa,  Fundação de Medicina Tropical,  Dr Heitor Vieira Dourado, Manaus, Brazil and Elisa Vidal, MMV). |
|  |  |  |  | Deficient:  < 30%  enzyme activity | PQ 0.75 mg/kg over 8 weeks | Under medical supervision in areas with access to tertiary care |  |
|  |  |  |  | If no  testing available | PQ 0.5 mg/kg/day over 7 days | Advise patients to monitor for clinical symptoms and referral to reference hospital if suspected G6PD deficient or adverse events. |  |
|  |  |  |  | Normal:  > 70%  enzyme activity | TQ 300 mg gradually being implemented |  |  |
| Colombia [117] | 2022 | Recommended but test type not specified  For females qualitative testing recommended | Not  specified | Normal:  80%  activity | PQ  0.25–0.5 mg/kg over 14 days | Not specified | STANDARD G6PD rollout in context of PAVE studies  (Personal communication - Elisa Vidal, MMV) |
|  |  |  |  | Intermediate:  30–80% activity | Not  specified | Not specified |  |
|  |  |  |  | Deficient:  < 30%  activity | PQ 0.75 mg/kg over 8 weeks | With medical supervision and access to transfusion services |  |
|  |  |  |  | If no testing  available | PQ 0.25 mg/kg over 14 days based on risk-benefit assessment | With medical supervision and access to transfusion services |  |
| Costa Rica [118] | 2020 | Not explicitly  mentioned | Not  specified | Normal  (no definition) | PQ 0.25 mg/kg over 14 days or  0.5 mg/kg over 7 days if adherence is expected to be low | Counselling for adverse events | Unable to ascertain information on  implementation |
|  |  |  |  | Deficient:  < 30%  enzyme activity | PQ 0.75 mg/kg over 8 weeks | Not specified |  |
| Ecuador [119] | 2019 | Not explicitly  mentioned | Not  specified | Normal  (no definition) | 0.5 mg/kg over 7 days | Not specified | Unable to ascertain information on  implementation |
|  |  |  |  | Deficient  (no definition) | PQ 0.75 mg/kg over 8 weeks | With medical supervision |  |
|  |  |  |  | If no testing  available | Based on risk-benefit assessment | Not specified |  |
| French Guiana [120] | 2015 | Required,  central laboratory  diagnosis | Not  specified | Normal  (no definition) | PQ 0.25 mg/kg over 14 days | Not specified | Unable to ascertain information on  implementation |
|  |  |  |  | Moderate deficiency  (no definition) | PQ 0.75 mg/kg over 8 weeks |  |  |
| Guyana [121] | 2015 | Unclear,  only mentioned in  context of malaria in pregnancy | Not  specified | Normal  (no definition) | PQ 0.25 mg/kg over 14 days | Not specified | Unable to ascertain information on  implementation |
|  |  |  |  | Deficient  (no definition and only mentioned in context of malaria in pregnancy) | PQ 0.75 mg/kg over 8 weeks | With close medical supervision and access to transfusion services |  |
|  |  |  |  | If no testing  available  (only mentioned in context of malaria in pregnancy) | Based on risk-benefit assessment | Not specified |  |
| Honduras [122] | 2018 | Not explicitly  mentioned | Not  specified | Normal  (no definition) | PQ 0.25 mg/kg over 14 days or  0.5 mg/kg over 7 days at discretion of physician | Directly Observed Therapy | Unable to ascertain information on  implementation |
|  |  |  |  | Deficient  (no definition) | PQ 0.75 mg/kg over 8 weeks | Not specified |  |
|  |  |  |  | If no testing  available | Based on risk-benefit assessment | Not specified |  |
| Mexico [123] | 2022 | Not required  (not explicitly  recommended) | NA | Normal  (no definition) | PQ 0.5 mg/kg over 7 days | Not specified | Unable to ascertain information on  implementation |
|  |  |  |  | Deficient  (no definition) | PQ 0.75 mg/kg over 8 weeks |  |  |
|  |  |  |  | If no testing  available | PQ 0.25 mg/kg over 14 days |  |  |
| Nicaragua [124] | 2022 | Recommended,  qualitative | Not  specified | Normal:  > 30  enzyme activity | PQ 0.25 mg/kg over 14 days or 0.5 mg/kg over 7 days.  If frequent relapse PQ 0.5 mg/kg over 14 days | With counselling on adverse event for female patients | Unable to ascertain information on  implementation |
|  |  |  |  | Deficient:  < 30%  enzyme activity | PQ 0.75 mg/kg over 8 weeks | With close medical supervision and access to transfusion services |  |
|  |  |  |  | If no testing  available | Based on risk-benefit assessment | Not specified |  |
| Panama [125] | 2022 | Not explicitly  mentioned | Not  specified | Normal  (no definition) | PQ 0.25 mg/kg over 14 days  OR  PQ 0.5 mg/kg over 7 days  in areas with low G6PD deficient prevalence + poor adherence to 14-day regimen | Not specified | Unable to ascertain information on  implementation |
|  |  |  |  | Deficient  (no definition) | PQ 0.75 mg/kg over 8 weeks | Only with close medical supervision and access to transfusion services |  |
|  |  |  |  | Severely Deficient (no definition) | No PQ | NA |  |
|  |  |  |  | If no testing  available | PQ 0.25 mg/kg over 14 days. | With counselling for adverse events, pharmacovigilance, reinforcement of health services’ ability to adequately manage adverse events. |  |
| Peru [126] | 2015 | Not explicitly  mentioned | Not  specified | NA | PQ 0.5 mg/kg over 7 days | Not specified | SD Biosensor use planned in context of PAVE studies in 2023.  (Personal communication –  Dionicia Gamboa,  Institute of Tropical Medicine Alexander von Humboldt, Peru  and Elisa Vidal, MMV) |
| Suriname [127] | 2018 | Not explicitly  mentioned | Not  specified | NA | PQ 0.5 mg/kg over 14 days | Be aware of adverse events if patient G6PD deficient | SD Biosensor introduced in 2022 for high-risk localities and active screening for Amerindian tribal population.  G6PD testing to be required for TQ after introduction.  (Personal communication –  Dr. Helene Hiwat,  Coordinator Malaria Program, Suriname) |
| Venezuela [128] | 2017 | Not explicitly  mentioned  (recommended for 0.5 mg/day over 7 days) | Not  specified | Normal  (no definition) | PQ 0.25 mg/kg over 14 days  PQ 0.5 mg/day over 7 days with special  authorisation | Not specified | Unable to ascertain information on  implementation |
|  |  |  |  | Mild-moderate (no definition) | PQ 0.75 mg/kg over 8 weeks | Under medical supervision |  |
|  |  |  |  | Severe  (no definition) | No PQ | NA |  |

Treatment guidelines could not be found for Guatemala. NA = Not applicable
