# Supplementary material for: A Review of the Current Status of G6PD Deficiency Testing to Guide Radical Cure Treatment for Vivax Malaria
Source: Pathogens. 2023 Apr 27;12(5):650. doi: 10.3390/pathogens12050650 (PMC10220632; doi:10.3390/pathogens12050650)
Supplement: Supplementary file 1 [file pathogens-12-00650-s001.zip › pathogens-2342386-supplementary/G6PD Dx Rev_suppl_Text S1.docx]

Supplementary Information to A review of the current status of G6PD deficiency testing to guide radical cure treatment for vivax malaria

**Text S1:** Methodology for policy review

**Inclusion of countries:** Countries reporting endemic vivax cases in 2020 as per World Malaria Report [3]

**Sourcing of documents:** National treatment guidelines and national strategic plans were sourced online and through direct contact with national malaria control programs or partners working in the respective countries. Where documents could not be sourced, this is indicated.

**Data extraction:** Data was extracted for the following predefined themes: policy recommendation on G6PD testing, definition of 100% enzyme activity, categorization of G6PD status, policy recommendation on hypnozontocidal treatment, and any additional recommendation to ensure treatment safety. Data extraction was done by two authors independently and compared and discussed in the group in case of discrepancy. Non-English guidelines were translated by collaborators whenever possible.

**Implementation status:** For the Asia-Pacific countries information on current practice of G6PD testing was obtained from National Malaria Program Representatives attending the Vivax Working Group Meeting of the Asia Pacific Malaria Elimination Network in December 2022 and followed up with individuals if required. For the other countries authors reached out to their professional networks.
